# Clopidogrel-Induced Severe Hepatitis: A Case Report and Literature Review

**DOI:** 10.1155/2016/8068276

**Published:** 2016-06-27

**Authors:** Hesam Keshmiri, Anuj Behal, Shawn Shroff, Charles Berkelhammer

**Affiliations:** Department of Medicine, Advocate Christ Medical Center, University of Illinois, Oak Lawn, IL 60453, USA

## Abstract

Clopidogrel is a commonly prescribed antiplatelet agent that carries a rare risk of hepatotoxicity. We describe a case of severe clopidogrel-induced hepatitis with liver biopsy assessment. Prompt recognition and withdrawal of the offending agent are imperative to prevent progression and potentially fatal liver injury.

## 1. Introduction

Clopidogrel is a commonly used antiplatelet agent, yet only several cases of hepatotoxicity have been described [1–16]. Liver biopsies were not performed in many of these cases. We report a rare case of severe clopidogrel-induced hepatitis with histological assessment.

## 2. Case Description

A 34-year-old male with a history of coronary artery disease and remote coronary artery stent was placed on aspirin plus clopidogrel. His baseline liver biochemistries were normal. He had been on clopidogrel for 2 months 12 years ago without adverse effects but discontinued the medication on his own at that time due to nonadherence. Four and a half months after restarting clopidogrel, he presented with jaundice and fatigue. He denied fever, rash, arthralgias, or abdominal pain. His only other medications were aspirin and metoprolol, which he had been on for many years with normal liver biochemistries.

The patient was not on a statin. He denied recent alcohol or herbal medications. Physical examination was significant only for icterus. There was no hepatosplenomegaly, clubbing, rash, asterixis, or other stigmata of chronic liver disease.

Initial bilirubin was 5.7 mg/dL (normal 0.2–1.2 mg/dL), ALT 1,393 U/L (normal 7–48 U/L), AST 1,418 U/L (normal 7–48 U/L), alkaline phosphatase 130 U/L (normal 35–115 U/L), INR 1.5, and partial prothrombin time 37 seconds (normal 15–37 seconds). Extensive serologies were negative to hepatitis A, hepatitis B, hepatitis C (including hepatitis C RNA), hepatitis E, IgM to cytomegalovirus and Epstein-Barr virus, anti-nuclear antibody, anti-smooth muscle antibody, anti-mitochondrial antibody, anti-liver kidney microsomal antibody, and ceruloplasmin.

**Figure 1:**
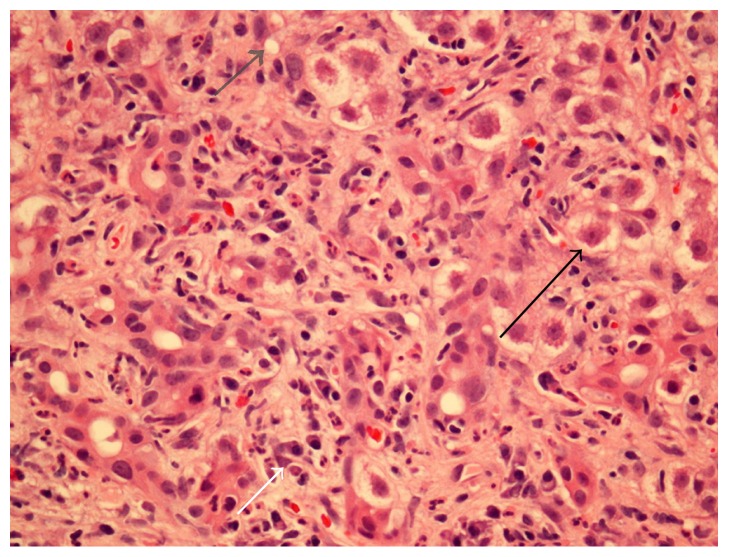
Liver biopsy showing severe acute hepatitis with mixed inflammatory portal tract infiltrate (white arrow), patchy hepatocyte ballooning degeneration (black arrow), and extensive periportal hepatocyte dropout (grey arrow).

Imaging studies were negative, and bile ducts were not dilated, including by ultrasound, computed tomography, and endoscopic retrograde cholangiopancreatography. No gallstones were present on any imaging modality. Liver biopsy revealed severe acute hepatitis with mixed inflammatory portal tract infiltrates including plasma cells, neutrophils and eosinophils, bile ductular reaction, patchy hepatocyte ballooning degeneration, and extensive periportal hepatocyte dropout, without fibrosis (Figure 1).

The patient was diagnosed with clopidogrel-induced severe hepatitis. Despite discontinuing clopidogrel, AST increased to 2,107 U/L, ALT to 1,567 U/L, and bilirubin to 37 mg/dL (predominately direct bilirubin). INR had increased to 2.1 despite empiric administration of vitamin K. A brief course of prednisone and ursodiol was initiated, with subsequent normalization of liver biochemistries.

## 3. Discussion

We describe a rare case of severe clopidogrel-induced hepatitis, with histological assessment. Our patient's drug-induced hepatitis was particularly severe with jaundice (peak bilirubin 37 mg/dL), marked elevation of transaminases (peak ALT of 1,567 U/L), and coagulopathy (INR 2.1). This degree of hepatic injury portends an increased mortality and underscores the importance of early recognition and discontinuation of the offending agent.

Clopidogrel-induced hepatitis has been described [1–16].  Table 1 lists reported cases in reverse chronological order. The degree of liver injury has ranged from reversible liver injury and recovery [1–9, 11–16] to acute hepatic failure and death [10]. Onset of liver injury in these cases ranges between 3 and 180 days [1–16]. Rechallenge confirmed clopidogrel-induced hepatitis in some of these cases [2–5]. Our patient's Naranjo scale and RUCAM (Roussel Uclaf Causality Assessment Method) scores were both 8, indicating probable drug-induced hepatitis [17, 18].

Our patients liver biopsy revealed severe hepatocellular injury. This adds to the histological findings in clopidogrel-induced hepatitis, as liver biopsy was only performed in 3 of the previously reported cases [1, 2, 11]. Clopidogrel-induced liver injury can be cholestatic, hepatocellular [11], or mixed hepatocellular plus cholestatic [1, 2].

The exact mechanism of clopidogrel-induced hepatitis is unclear. The delayed onset of 4.5 months in our case suggests a toxic-metabolic etiology, whereas the inflammatory infiltrate and response to corticosteroids raise the possibility of a superimposed immune mediated mechanism of injury. Clopidogrel is a prodrug which is metabolized to inactive clopidogrel carboxylate (90%) and an active metabolite containing a mercapto group (10%) by cytochrome P450 3A4 and 2C19. In vitro studies suggest that the active metabolite is responsible for the hepatotoxicity and that high cytochrome 3A4 activities coupled with cellular glutathione depletion are potential risk factors [19]. Interestingly, an earlier antiplatelet agent, ticlopidine, has also been reported to cause drug-induced cholestatic hepatitis [20, 21].

Clopidogrel-induced hepatitis is a rare but potentially serious adverse effect. A high degree of clinical suspicion is required in patients presenting with abnormal liver biochemistries within a few months after starting clopidogrel. Prompt recognition and discontinuation of the offending agent are necessary, as progressive liver injury and even death can occur.

## Figures and Tables

**Table 1:** Clopidogrel-induced hepatitis: reported cases.

Cases	Latency/onset	Peak ALT (U/L)	Peak bilirubin (mg/dL)	Peak alkaline phosphatase (U/L)	Symptoms	Histology	Outcome
Keshmiri et al. 2016 (current)	135 days (4.5 months)	1567	37	130	Jaundice, fatigue	Hepatocellular	Recovery
Kapila et al. 2015 [9]	5 days	716	1.6	160	Nausea, vomiting, fever	No liver biopsy	Recovery
Pisapia et al. 2015 [2]	3 days	1603	24.5	408	Jaundice, arthralgia, papular rash	Mixed hepatocellular and cholestatic	Recovery
Monteiro et al. 2011 [13]	30 days	540	3.5	139	Nausea, vomiting	No liver biopsy	Recovery
Leighton et al. 2011 [11]	1 day	3626	3.3	364	Anorexia, jaundice, epigastric pain	Lymphoplasmacytic portal, interface and lobular hepatitis with no fibrosis	Recovery
Kastalli et al. 2010 [10]	19 days	336	4.7	186	Jaundice, abdominal pain	No liver biopsy	Death
Goyal et al. 2009 [3]	23 days	1011	7.3	1011	Abdominal pain, anorexia, jaundice	No liver biopsy	Recovery
Wiper et al. 2008 [4]	60 days	450	Normal	680	General malaise	No liver biopsy	Recovery
L$\overset{´}{o}$pez-Vicente et al. 2007 [12]	30 days	204	1.0	682	Abdominal pain, fever	No liver biopsy	Recovery
Ng et al. 2006 [5]	3 days	536	1.5	247	Fever, chills	No liver biopsy	Recovery
H$\ddot{o}$llm$\ddot{u}$ller et al. 2006 [1]	43 days	1003	20.8	221	Weight loss, anorexia, jaundice, nausea, abdominal pain	Mixed hepatocellular and cholestatic	Recovery
Chau et al. 2005 [7]	37 days	253	6.9	172	Jaundice	No liver biopsy	Recovery
Beltran-Robles et al. 2004 [6]	4 days	318	0.51	100	No symptoms mentioned	No liver biopsy	Recovery
Wolf et al. 2003 [16]	12 days	173	1.3	132	Fever, leukopenia, weakness	No liver biopsy	Recovery
Ramos Ramos et al. 2003 [14]	60 days	710	24.9	118	Jaundice	No liver biopsy	Recovery
Duran-Quintana et al. 2002 [8]	180 days	786	13	474	Icterus, hepatomegaly	No liver biopsy	Recovery
Willens 2000 [15]	21 days	507	3.0	636	Malaise, anorexia, myalgia, icterus	No liver biopsy	Recovery
